# Comparison of Emotional Behaviour of Spanish, Chilean and England Adolescents, and Their Relationship with Effective Personality

**DOI:** 10.3390/ijerph18168611

**Published:** 2021-08-15

**Authors:** María Eugenia Martin-Palacio, Andrés Fernando Avilés-Dávila, Cristina Di-Giusto, José-Antonio Bueno-Álvarez, Marta Soledad García-Rodríguez, Jesus Manuel Cedeira-Costales

**Affiliations:** 1 Department of Psychology and Research in Education, Faculty of Education and Teacher Training, Complutense University of Madrid, 28040 Madrid, Spain; andresav@ucm.es (A.F.A.-D.); alvarez@edu.ucm.es (J.-A.B.-Á.); 2 Department of Educational Sciences, Faculty of Education, University of Burgos, 09001 Burgos, Spain; cdi@ubu.es; 3 Department of Educational Sciences, Faculty of Teacher Training and Education, University of Oviedo, 33005 Oviedo, Spain; martagar@uniovi.es; 4 Department of Psychology, Faculty of Psychology, University of Oviedo, 33003 Oviedo, Spain; jesmac@telecable.es

**Keywords:** behaviour, effective personality, adolescents, gender, aggressive

## Abstract

Analysing the emotional behaviour of adolescents is fundamental because of its relationship with maladaptive behaviour and even possible psychological maladjustments. For this reason, this study had two objectives: to analyse the existence of significant differences in socio-emotional behaviour in English, Spanish, and Chilean adolescents, taking gender into account, and to analyse the relationship between emotional behaviour and the effective personality model in the Spanish and Chilean samples. A total of 2534 adolescents participated (609 English, 1677 Spanish, and 248 Chilean). The Abbreviated Scale of Emotional Behaviour (ECEA_R: aggressive tendency, social reactivity, and social support) and the Effective Personality Questionnaire—Adolescents (CPE-A: academic self-realisation, socio-affective self-realisation, and resolute efficacy) were applied. A MANOVA was carried out to study the differences in adolescents’ socio-emotional behaviour, taking gender and nationality into account, and a correlational analysis was undertaken to explore the relationship between the variables of emotional behaviour (aggressive tendency, social reactivity, and social support) and effective personality (academic self-realisation, socio-affective self-realisation, and resolute efficacy). Regarding the first objective, for aggressive tendency, English male adolescents stood out, followed by Spanish and Chilean male adolescents and females of all nationalities. In terms of social reactivity, female adolescents stood out over male adolescents and, with regard to social support, Spanish adolescents (male and female) stood out over other nationalities, followed by Chilean and English adolescents (males and females). The results of the second objective indicated a negative relationship between aggressive tendency and academic self-realisation, but a positive relationship for social reactivity and social support (only in the Spanish sample) with most of the effective personality factors. The results are relevant for the application of prevention and intervention programs that improve or implement social and affective competencies in adolescents who develop the effective personality construct.

## 1. Introduction

Analysing adolescent emotional behaviour is essential for understanding the relationship it has with the appearance of maladaptive behaviours and even possible psychological maladjustments [1]. However, interest in emotional behaviour research is relatively recent: some authors have pointed out that there has been a striking paucity of research at the national and international levels [2]. In Spain, before this date, the most frequent and abundant studies have focused on aggressive behaviour [3] and the relationship between being a victim of bullying and being an aggressor, alongside different parameters associated with emotional intelligence [4]. At the international level, up to 2014 [2], only four international studies had been published on this subject: two conducted with primary school children—one in the United States [5] and another in England [6]—and two conducted with samples of adolescents, one in Germany [7] and another in China [8].

This apparent lack of interest in research on emotional behaviour is striking, given its importance for the adequate adaptation of human beings to different developmental contexts, and for the psychological adjustment of children and adolescents [9].

Fortunately, in recent years, the importance of studying emotional behaviour in adolescents has been reflected in the increase in research on emotional behaviour in general, both nationally and internationally [10,11,12,13]. Research in Spain has mostly focused on violent, aggressive, and risky behaviour in schools and its influence on the quality of the teaching–learning process [14,15], on the link that these behaviours have with possible lesions in the prefrontal cortex [16], or on the influence that the use of new technologies has on these behaviours [17].

An analysis of the relevant literature has also noted the scarcity of cross-cultural studies on the manifestations of emotional behaviours in adolescents, which is striking, given that the importance of the influence that the social and cultural environment and the characteristics of the context have on behaviour has been demonstrated [12]. Despite this, some studies have been found, such as that of Fernández [18], who compared British and Spanish samples, and that of Muñoz [19], who analysed Chilean and Spanish samples, and the relationships of these behaviours with the dimensions of the effective personality construct [20].

Evaluating emotional behaviour will facilitate the generation, implementation, and evaluation of prevention and intervention programs that improve the social and affective competencies of adolescents for regulating adaptive emotional behaviour in all contexts. It is also important to note that cross-cultural studies offer a broader vision that will allow programs to be designed and adapted appropriately to different populations.

Analysing this behaviour in terms of the gender variable is fundamental [21,22] because gender stereotypes are still in force among the adolescent population, despite the social changes of recent decades. Furthermore, taking the differences in emotional behaviour derived from stereotypes into account is considered to be very important when designing educational interventions.

The effective personality construct, developed by Martín Del Buey, Martín Palacio, and their research group [20,23], defines effective personality as follows: “An effective person is a living being with knowledge and self-esteem (self-concept and self-esteem) in a process of constant maturation (at any stage of its evolution) with the capacity (intelligence) to achieve (effectiveness) what he/she desires (motivation) and expects (expectation) using the best possible means (training) (efficiency), controlling the causes (attribution of causality) of their achievement (success or failure) by facing the personal, circumstantial and social difficulties (coping with problems) that arise, making the right decisions without detriment to their good relations with others (empathy and communication) or renouncing their fair personal aspirations (assertiveness).” [20] (p. 121).

If we adapt this definition to the adolescent field, a pupil who possesses certain traits considered to be typical of an effective personality in the various psychosocial contexts in which they participate would be characterized by: a broad knowledge of self (self-concept) and an adequate assessment of self (self-esteem), the motivation to study and to face the challenges that the educational field offers (motivation), a hope of achieving what they set out to do (expectations), constant evaluation of the causes of what is happening to them (attributions), the wisdom to deal effectively with the impediments that arise when trying to achieve what they are motivated to do (coping with problems) and to choose the best option among the many alternatives involved in the various decisions (decision-making), and the ability to coexist in the social environment in which they interact with others, knowing how to express their opinions and feelings without offending others (assertiveness, empathy and communication) [24].

The study by Fernández [18] addressed the relationships between the constituent variables of the effective personality construct and certain adolescent emotional behaviours, and compared, in one section of the study, the differences and similarities between British and Spanish adolescents. 

Another study of reference in our work is that of Muñoz [19], who proposed a similar study in Chilean adolescents, also within the framework of the effective personality construct, using the same measurement instruments and methodology of analysis as Fernández [18]. The latter study also presented a comparative analysis of similarities and differences between Chilean and Spanish adolescents. It also provided the first approach to undertaking a comparative analysis among the three samples studied: British, Chilean, and Spanish.

Other cross-cultural studies which relate certain emotional behaviours and an effective personality [18,19] can be considered as a precedent for the present study. These include the research conducted in Spain by Romero and Alonso [25], which examined the relationship between the five major maladaptive traits included in the DSM-5 with multiple adolescent emotional behaviours, and the study conducted by Guasp Coll et al. [26], which studied the importance of sociodemographic variables (gender and age), empathy, and emotional intelligence (EI) on self-esteem and life satisfaction. This study presented the correlations found between the three dimensions of emotional behaviour studied and dimensions corresponding to the effective personality construct. Adolescent emotional development, regulation, and behaviour can be interpreted more broadly within the framework of the effective personality construct in the sense that certain variables of efficacy appear as descriptors in the emotional behaviour scales and coincide with the factors present in the effective personality construct.

The exploratory instruments used in the studies by both Fernández [18] and Muñoz [19] included an abbreviated version of the Emotional Behaviour Scale (EBS) [27], which was used by Fernández [18] for secondary school students; The Emotional Behaviour Scale for Adolescents Revised (ECEA-R) and the abbreviated version of the Questionnaire of Effective Personality in Adolescent Contexts (CPE-A) by Martín del Buey et al. [28]. In both versions and during the adaptation processes, the criteria established by Muñiz and Hambleton [29] and Muñiz et al. [30] were followed. 

The present work, compared with previous studies, represents an advance for two main reasons. Firstly, it is a cross-cultural study on adolescent emotional behaviour in samples from three countries, taking the gender variable into account. Secondly, unlike others, it analyses the relationship between effective personality factors and emotional behaviour in Latin samples. Therefore, the study had two objectives: on the one hand, it aimed to analyse the existence of significant differences in emotional behaviour among adolescents of three nationalities, taking the gender variable into account; on the other hand, it aimed to analyse the relationship between emotional behaviour and the dimensions of the effective personality construct in the Spanish and Chilean samples. The reason for not applying the effective personality questionnaire to the English sample is that there was no adaptation of the instrument to that population. The hypothesis related to the first objective is that there will be statistically significant differences according to nationality and gender. More specifically, English males will have statistically higher scores for aggressive tendency, and Spanish females will have higher scores for social reactivity and social support. With respect to the second objective, the hypothesis is that there will be positive correlations between the effective personality construct and the social support and social reactivity factors, but negative correlations with the aggressive tendency factor.

## 2. Materials and Methods

To achieve the objectives and confirm the hypotheses, a non-experimental quantitative methodology was used that applied self-report questionnaires.

### 2.1. Participants

The population under analysis comprised adolescents from England, Spain, and Chile, where according to census data, those aged 15–24 years old totalled 7,622,161 (11.71% of the U.K. population), 4,758,009 (9.65% of the Spanish population), and 2,623,177 (14.63% of the Chilean population), respectively [31]. For the first objective, a sample of 2534 adolescents was selected through non-probabilistic convenience sampling, of which 609 were English from York County, 1677 were Spanish from six Autonomous Communities, and 248 were Chileans from the V Region. All the results found had a confidence level of 95% and a margin of error of 4%, 2.4%, and 6.3% respectively. The inclusion criteria were: enrolment at school; being in compulsory secondary education or its equivalent, depending on the country; and having the informed consent form signed by parents or guardians. Exclusion criteria were a refusal to complete the questionnaire or a diagnosis of intellectual disability. Table 1 shows the most representative characteristics of each of the samples by country. It can be seen that both England and Spain were fairly homogeneous concerning the gender variable, but this was more unequal in the Chilean sample. Regarding the academic years, it should be noted that there were no English participants in the 15–16-year-old age group and there was a slight imbalance in the Chilean students in the first and second years of secondary school.

For the second objective, we used the total Chilean sample (the 248 adolescents described above) and a subsample of Spanish adolescents (151 students from two schools in the north of Spain) to whom we applied the Emotional Behaviour and Effective Personality scales. The effective personality test was not applied to all of the Spanish sample, so only a subsample of it was used, nor to the English sample because no adaptation of the instrument to that population was available.

### 2.2. Measures

Two instruments were used, one for assessing adolescent emotional behaviour and the other for assessing effective personality.

The Abbreviated Scale of Emotional Behaviour (ECEA-R) [18] is the Spanish version of the reduced Emotional Behaviour Scale (EBS) [27]. The ECEA-R is answered on a dichotomous scale (yes = 1 and no = 0) and consists of 36 items grouped into 3 factors:

- Aggressive tendency consists of 12 items with a Cronbach’s alpha reliability of 0.748. It assesses difficulty in controlling aggressive behaviour, acting impulsively, the desire for revenge, hostility, and having little regard for the feelings of others.
- Social reactivity consists of 15 items with a Cronbach’s alpha reliability of 0.759. It studies the extent to which adolescents can put themselves in the place of others, worry easily, and ruminate on their problems. They show prosocial (helping others) behaviours and easily feel guilty or worried if they believe they have hurt others’ feelings or have failed them.
- Social support has 9 items with a Cronbach’s alpha reliability of 0.704. It analyses adequate social competence and having a support network that allows students to feel safe and supported by their peers. They maintain a perception of affection and the availability of relevant attachment figures at the interpersonal level.

The Abbreviated Effective Personality Questionnaire for Secondary Education [28] is answered on a Likert scale of 5 possible scores (1 = strongly disagree to 5 = strongly agree) and consists of 28 items distributed in 3 subscales:

- Academic self-realisation has 11 items and presents a Cronbach’s alpha reliability of 0.861. This factor comprises the variables that support effective execution of the academic component. Items measuring indicators related to self-concept, self-esteem, motivation, expectations, and academic attributions of success are included here. Therefore, students who score high on this factor would value themselves positively as students, their motivations for studying would be mainly internal, and they would be oriented towards learning new things, testing their ability, overcoming challenges, and exercising autonomy and personal control. They also attribute their success to their ability and effort, and have expectations of success, both near and far.
- Socio-affective self-realisation consists of 11 items with a Cronbach’s alpha reliability of 0.796. These factor group variables that support the effective performance of the socio-affective component. Items measuring indicators related to self-concept, self-esteem, attributions, expectations, and successful relationship skills are combined here. Therefore, a student who scores high on this factor would have a good knowledge and appreciation of their physique and relationships, would show a high level of self-esteem and self-confidence at both the personal and social levels, would attribute their social relationships to their ability to relate and would have good communication skills, as well as expectations of success in their relationships.
- Resolute efficacy, with 6 items, has a Cronbach’s alpha reliability of 0.768. This factor incorporates variables that support effective coping with challenges that may arise in all domains. Items measuring indicators related to decision making and coping with problems are combined here. Thus, a student who scores high on this factor would carefully plan the decisions they have to make (whatever they may be) by adjusting to the demands of each situation, gathering as much information as they can find, and analysing the possible consequences of their decisions. Moreover, they would deal positively with problematic situations, displaying optimism, perseverance, and the ability to learn from experience.

### 2.3. Procedures

Firstly, contact was made with the different schools, informing them by e-mail of the project to be carried out and asking for their collaboration. Once the activity had been approved by the school management, a member of the research team met with the teachers responsible to set the dates and times for administering the questionnaires. In addition, at the meeting, informed consent forms were handed out for teachers to send to the students’ legal guardians to be signed before the questionnaires were administered. The questionnaires were administered in the classrooms of the schools in the 3 countries, with the prior informed consent of those responsible for the schools and the students’ legal guardians. The Spanish and Chilean samples were collected by members of the research team, while the English sample was collected by Dr. Jane Clarbour’s team at the University of York, as part of the process of studying and validating the original version of the EBS. The Chilean students were administered the Emotional Behaviour and Effective Personality scale, as were a subsample of Spanish students, while the others were administered only the first questionnaire. The questionnaires were administered collectively and voluntarily, ensuring the confidentiality and anonymity of the information collected at all times, and clearly stating that the results would be treated as statistics. The questionnaire application procedure was carried out in a single session per classroom during school hours. The administrator read the instructions at the top of the protocol and checked that the participants understood how to answer the questionnaire. Students were instructed to try to be as honest as possible and were informed that there were no correct or incorrect answers. In the event of any incident or doubt, the students were attended to individually.

### 2.4. Data Analysis

To achieve the first objective, a multivariate analysis of variance (MANOVA) was carried out to determine whether there were statistically significant differences according to the country and gender of the participants regarding emotional competencies. If the results were significant, the MANOVA was completed with an analysis of variance (ANOVA) or Student’s t-test and the relevant post hoc tests. Moreover, in all cases, the effect size was analysed using the Partial Eta Squared Coefficient to determine the significance of the differences found. Concerning the second objective, to determine the relationship between emotional behaviour and effective personality, a correlational study was carried out using Spearman’s correlation. All the analyses were performed using the SPSS 25.0 statistical software.

## 3. Results

To achieve the first objective of finding out the differences according to country and gender in the emotional competence factors, a study of differences was carried out using a MANOVA. To verify the assumptions of normality of the MANOVA, the criterion of Pérez [32] was taken into account, which considers a population to be normal if the sample presents skewness and kurtosis coefficients of ± 2. Table 2 shows that the coefficients are within this interval in each of the study groups. It was therefore considered that the normality criterion was adequately met, in addition to the guarantee of being large groups (*n* > 30), in which the absence of normality does not affect the sampling distribution of the statistic. The assumption of multicollinearity was also met, finding low correlations among the three dependent variables, which ranged between 0.154 and 0.202; the VIFs ranged between 1.001 and 1.043; and the eigen values were all less than 10. All of these are considered suitable values for MANOVA and ANOVA analyses according to the scientific literature [33].

To understand the level of emotional competence of each of the groups, Table 3 presents the descriptive statistics for each of them regarding the factors in the questionnaire. It can be seen that males in all three countries had higher scores for aggressive tendency and social support, whereas females stood out for social reactivity. The English students also had higher scores than the other countries for aggressive tendency, while the Spanish sample had the highest scores for social support and social reactivity.

To determine whether these differences were statistically significant, the multivariate Pillai trace statistic was chosen as one of the most robust and powerful statistics [34]. It was found to be statistically significant for the comparison between countries (F = 41.509, *p* = 0.000, η_p_^2^ = 0.047), between genders (F = 150.975b, *p* = 0.000, η_p_^2^ = 0.152), and for the country × gender combination (F = 2.893, *p* = 0.000, η_p_^2^ = 0.003), but the effect size was low except for gender, which was high [35].

Next, independent ANOVAs were performed, but as Levene’s test indicated heteroscedasticity (*p* < 0.05), a heteroscedastic ANOVA was performed using Welch’s correction (Welch test) for the analysis of variables with more than two levels; Student’s *t*-test, not assuming equal variances where appropriate, was used for gender. 

Table 4 shows statistically significant differences in all the dependent variables for both country and country × gender, where it was necessary to carry out post hoc tests to determine which groups these differences occurred between. Concerning the gender variable, statistically, significant differences were observed in the three factors, with males scoring higher for aggressive tendency and social support, but lower for social reactivity. All effect sizes were low, except for social support as a function of country and social reactivity as a function of gender, for which they were medium.

When the contrast tests were carried out for the variable country (Table 5), it could be seen that there were differences among the three countries for aggressive tendency, with England having the highest values and Chile the lowest; for aggressive tendency and social support, differences were found between Spain (with higher values) and England and Chile, with these differences not being significant between England and Chile.

The differences, analysed in more detail for the country × gender variable, are shown in Table 6.

Generally speaking, for aggressive tendency, English and Spanish males had the highest scores (there were no significant differences between them, but there were differences with the rest of the groups). It is worth noting that males’ scores were higher than those of females in all the countries analysed.

For social reactivity, there were statistically significant differences among all the groups, except for English and Chilean males, who had the lowest scores for this factor, and there was also a correlation between the scores of females in these countries. The highest score for this factor was for Spanish females; it is worth noting that Spanish males also had higher scores than males from the other countries, but without reaching the level of the scores of females, who had higher scores.

For the social support factor, the Spanish samples were the ones with the highest scores, showing differences with the other groups. Even among the Spanish samples, it can be observed that the scores of the Spanish males were higher than those of the females. The English and Chilean samples were quite similar to each other, with differences found only between English males and Chilean females, the latter having the lowest scores for this factor. As for aggressive tendency, it can be noted that males’ scores were higher than females in all the countries analysed.

**Figure 1:**
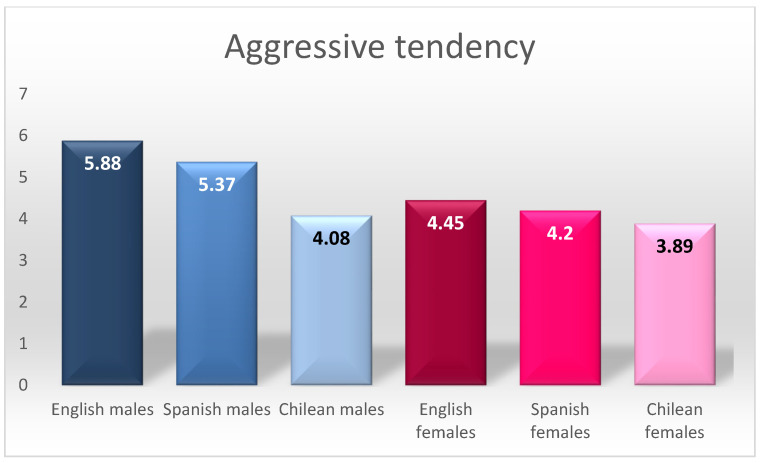
Summary of differences in aggressive tendency.

**Figure 2:**
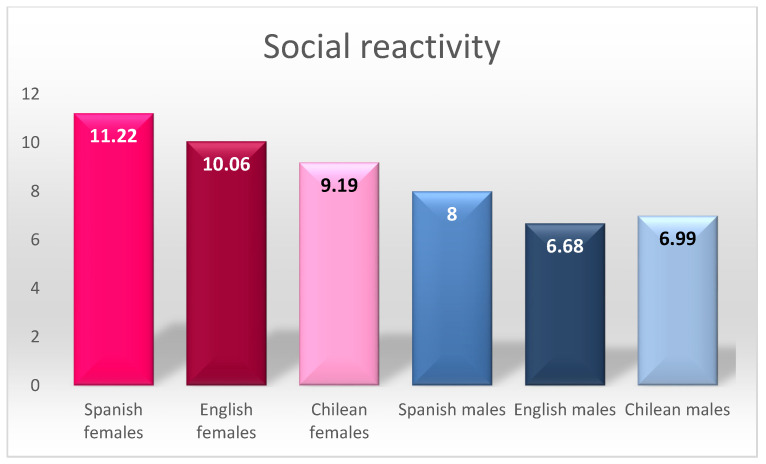
Summary of differences in social reactivity.

**Figure 3:**
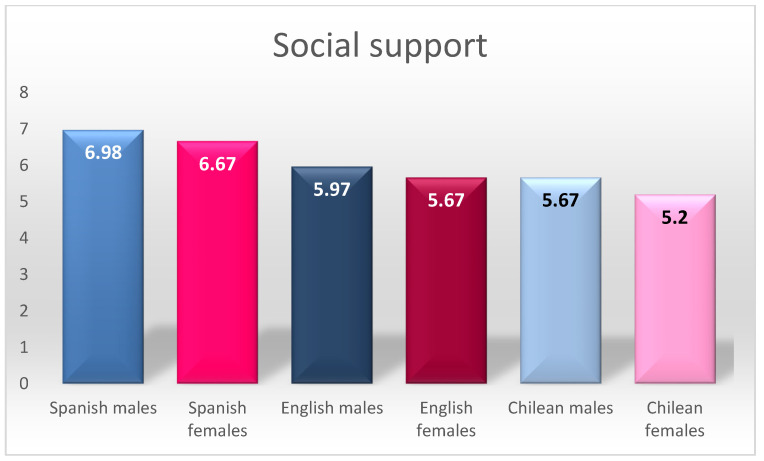
Summary of differences in social support.

By way of summary, Figure 1, Figure 2 and Figure 3 graphically present the differences found in aggressive tendency, social reactivity, and social support.

To achieve the objective of analysing the relationship between emotional behaviour and the dimensions of the effective personality model, a Spearman’s Rho correlation test was carried out between a subsample of 151 Spaniards and the 248 Chileans who were given both questionnaires. This test was applied after checking that the distribution was non-parametric using the Kolmogorov–Smirnov test (*p* < 0.05). Table 7 shows that the aggressive tendency factor correlated significantly and negatively with academic self-realisation in both samples, but with resolute efficacy only in the Spanish sample. The social reactivity factor correlated positively with all the factors of effective personality in the Chilean sample, but in the Spanish sample, this correlation was only observed for the factors academic self-realisation and socio-affective self-realisation, and there was no relationship with resolute efficacy. For social support, statistically significant positive correlations were found in the Spanish sample with the factors academic self-realisation and socio-affective self-realisation.

## 4. Discussion

This paper had two objectives: on the one hand, it aimed to analyse the existence of significant differences in emotional behaviour among adolescents of three nationalities (English, Spanish, and Chilean), taking the gender variable into account; on the other hand, it aimed to analyse the relationship between emotional behaviour and the dimensions of the effective personality model in a sample of Spanish and Chilean adolescents. 

Regarding the first objective, the hypothesis was that there would be statistically significant differences according to nationality and gender. More specifically, English males would have statistically higher scores for aggressive tendency, and Spanish females would have higher scores for social reactivity and social support. The results derived from the MANOVA analysis partially confirmed this hypothesis.

In terms of aggressive tendency, when we performed a combined analysis of nationality and gender, the results reflected two groupings, one formed by English and Spanish males, who had the highest scores for this factor, and another grouping formed by Chilean males and all the females in the samples from the different countries. The data from the English and Spanish samples confirmed previous findings regarding the higher overall prevalence of aggressive emotional expression in males [18,19,36,37,38,39,40]. However, they contradict the results found in the Chilean sample, where there were no such differences, as in the studies by Redondo Pacheco et al. [41].

On the other hand, the intercultural differences between Chile and the countries of England and Spain were predictable, based on knowledge of the influence of the social and cultural environment and the characteristics of the context on behaviour [12]. Some studies have confirmed these cultural differences in the expression of aggression depend on the country of origin, such as that of Torregrosa et al. [42], who analysed the differences in aggressive behaviour among Spanish, Chinese, and Mexican adolescents, or that of Li et al. [43], who pointed out differences concerning relational aggression between Chinese and American adolescents. Despite this, no intragender differences were found between English and Spanish adolescents, although there were intergender differences overall, which points to the existence of similar characteristics in aggressive tendencies in the countries of England and Spain. This could be explained by the fact that both are European countries in which competitiveness is reinforced, linked to traditional gender values where males have greater difficulties in controlling aggressive behaviour, acting more impulsively, showing a greater desire for revenge, showing hostility and showing less consideration for the feelings of others, while there were no such gender differences in Chileans, who showed greater control of aggressive impulses, taking the limits of adolescence into account.

When analysing the effect sizes of the differences, we found that they were low both by gender and by country, which is in line with studies such as that of Sanchis-Sanchis et al. [12] but differs from Archer [36], who found higher effect sizes. It is worth noting the problem that many of the studies did not show the effect size of the differences. 

When analysing the social reactivity factor in detail, we found four distinct groupings. The highest scores were obtained by Spanish females, followed by English and Chilean females, followed by Spanish males, and finally with the lowest scores for English and Chilean males.

This confirms previous findings regarding the relationship between empathy and gender in the sense that females express more internalizing emotions, both positive and negative [12,18,19,44,45], as well as the existence of a greater empathic disposition in females [46,47,48]. This may be due to the perpetuation of gender stereotypes that consider females to have a greater capacity to put themselves in the place of others, to show pro-social behaviour, to worry easily, to ruminate on problems, and to feel guilty or concerned about the feelings of others.

The differences found between Spaniards and the other countries are also in line with cross-cultural studies that found differences when analysing empathy, such as [49], who found differences between British and Venezuelan samples. However, as in the previous study, no differences were found between English and Chilean males or between English and Chilean females, which again points to the similarity between these two populations. 

In relation to this factor, the effect sizes for differences were low, except by gender, where they were moderate, which may indicate that this is more a matter of gender stereotypes or roles. This is in line with most studies [12,44,45,48] that found significant differences but low effect sizes by gender.

Analysing the social support factor, we found three groupings: the first one made up of the highest scoring Spanish males, the second made up of Spanish females and the third is made up of the rest of the participants; in the latter case, significant differences were found between English males and Chilean females. The gender differences in the Spanish sample could be explained on the basis that gender stereotypes are still prevalent among the adolescent population despite social changes [12,18,19,21,22], although these differences are not found in the English and Chilean samples. Concerning nationality, the Spanish sample showed higher indices for this factor. These results contradict the findings of the cross-cultural study by Serrano et al. [50] regarding the Spanish sample specifically, although their results support the similarity between Chileans and English, stating that Latinos have increasingly individualistic characteristics rather than more social and collective behaviours. 

It is worth mentioning that the effect sizes of the differences were low, except by country, where they were moderate. This may indicate that this is more a sociodemographic cultural issue than a gender issue. The low effect size was consistent with the study by Sanchis-Sanchis et al. [12] but the others did not report information on the effect size. 

To summarise the first objective, gender and cross-cultural differences were found for all the emotional behaviour variables. This could be explained based on studies such as that of Palacios and Martín [21], who stated that the context surrounding males and females influenced the origin of the differences, as well as the fact that gender differences may be due to the failure to overcome traditional cultural stereotypes. 

As for the second objective, to analyse the relationship between emotional behaviour (aggressive tendency, social reactivity, and social support) and the dimensions (academic self-realisation, socio-affective self-realisation, and resolute efficacy) of the effective personality model in a sample of Spanish and Chilean adolescents, the results support the hypothesis of the existence of positive correlations between the effective personality construct and the social support and social reactivity factors, and negative correlations with the aggressive tendency factor.

For aggressive tendency in the Chilean sample and the Spanish sample, there were negative correlations with academic self-realisation. This is because those students who presented higher scores in academic self-realisation presented lower aggressive tendencies. Moreover, at this age, school activities are one of the aspects that can frustrate them the most, generating a poor self-concept, so that one way of overcoming this could be aggressive tendencies. This indicates that adolescents who value themselves positively as students, whose motivations for studying are mainly of an internal nature and have attributions and expectations of success based on their ability and effort are the ones who show the least aggressive tendencies. Therefore, they are also characterised by controlling aggressive behaviour, acting reflectively, acting without a desire for revenge or hostility, and considering the feelings of others. Moreover, in the Spanish sample, aggressive tendency also correlated negatively with the resolute efficacy variable. These results are consistent with those reported by Torregrosa et al. [51], according to which, in most cases, students with highly aggressive behaviour were also more likely to be less interested in school subjects and to have more emotionally unstable behaviours than their non-aggressive peers. The results are also in line with research indicating that students with aggressive behaviour have lower self-esteem [52,53,54] and with the work of Esteve et al. [55], who found in their research that, the lower the self-concept scores, the higher the tendency to accept aggressive behaviour.

For the social reactivity factor, in the Chilean sample, there were significant correlations with all the effective personality factors (academic self-realisation, socio-affective self-realisation, and resolute efficacy). In the Spanish sample, there were only such correlations with the factors academic self-realisation and socio-affective self-realisation. These results are in line with those provided by Castro-Sánchez et al. [56], who found a positive and direct relationship between self-concept and cognitive empathy, and with the results of Usán and Salavera [57], who showed significant relationships between intrinsic school motivation and emotional intelligence, to a greater extent than with extrinsic motivation and lack of motivation. 

This implies that adolescents who show prosocial behaviour, can put themselves in the place of others, worry easily and ruminate on their problems (social reactivity), are also those who value themselves positively as students, whose motivations to study are mainly internal, and who have attributions and expectations of success based on their ability and effort (academic self-realisation). Furthermore, these adolescents had a high social and physical self-esteem attributed to their good interpersonal and communication skills, which they rely on for the creation of new social bonds (socio-affective self-realisation).

The Chilean sample was also characterized by planning their decisions, gathering information, adjusting to each situation, and analysing possible consequences in an optimistic, persevering, and resilient way. According to these results, young people who scored higher for academic self-realisation and socio-affective self-realisation (and resolute efficacy in Chile) also had higher scores for social reactivity.

Emotional clarity and emotional repair correlated positively with both self-esteem and life satisfaction [58].

This indicates that Spanish adolescents who plan their decisions, gather information, adjusting to each situation, and analyse the possible consequences in an optimistic, persevering, and resilient way are the ones with the least aggressive tendencies.

## 5. Practical Implications 

The analysis of emotional behaviour in adolescents makes it possible to generate personalised prevention and intervention programs that develop personal and socio-affective competencies, and the analyses of this study may allow them to be developed, taking cultural and gender differences into account. Given that a relationship has also been found with the variables of effective personality, programs that have already been created based on this model could be used with this objective to be implemented in different educational, institutional, and family settings from childhood onwards, preferably when the activities designed in them, adapted to the different ages, are included in the curricular content. It should be borne in mind that this implies the prior training, participation, and active involvement of teachers or guidance counsellors.

## 6. Limitations and Future Directions

The study has some limitations that should be considered when interpreting and generalizing the results. The first limitation refers to the sample, particularly the size decompensation of the different groups compared. The use of a bootstrapping method to compare group means using ANOVA and MANOVA or Student’s t-test could be considered in future studies. It could also be possible to have a random sample with a larger number of schools and subjects to ensure the representativeness of the results, especially in England and Chile. Secondly, it should be noted that the effect size of the differences found among the groups was low, so generalisations to the population should be made with caution. Finally, the limitation of not applying the effective personality questionnaire to the English sample was because no adaptation of the instrument for that population was available. The adaptation of the questionnaire to the English population is another pending task.

## 7. Conclusions

In conclusion, the most significant relationship between the factors of emotional behaviour and the components of effective personality in adolescence is that directly between social support and socio-affective self-realisation. Thus, the solidity of social support (a positive and supportive social support network) and socio-affective self-realisation increases.

Regarding the social support factor, no significant correlations were found between social support and effective personality in the Chilean sample, but significant correlations were found in the Spanish sample for social support and academic self-realisation and socio-affective self-realisation. Studies such as those by Rodríguez-Fernández et al. [59,60,61] supported the influence of social support on students’ school engagement, a variable that has been identified as a crucial element for psychosocial development and academic success [62]. Ramos-Díaz et al. [63] pointed out that interpersonal social skills and positive self-esteem are variables of adequate adjustment to manage the constant emotional demands faced by adolescents in and out of school more effectively.

Likewise, the variables measured for academic self-realisation and resolute efficacy constitute valid buffers against aggressive tendencies.

## Figures and Tables

**Table 1:** Descriptive data of the sample.

		Country		
		England N (%)	Spain N (%)	Chile N (%)
Gender	Males	308 (50.57%)	815 (48.60%)	153 (61.69%)
	Females	301 (49.43%)	862 (51.40%)	95 (38.31%)
	Total	609 (100%)	1677 (100%)	248 (100%)
Course	12–13 years (1º ESO)	215 (35.30%)	448 (26.71%)	66 (26.61%)
	13–14 years (2º ESO)	206 (33.83%)	405 (24.15%)	56 (22.58%)
	14–15 years (3º ESO)	188 (30.87%)	435 (25.94%)	100 (40.32%)
	15–16 years (4º ESO)	0 (0%)	389 (23.20%)	26 (10.49%)
	Total	609 (100%)	1677 (100%)	248 (100%)

**Table 2:** Skewness (As) and kurtosis (k) indices.

Country	Gender	N	Aggressive Tendency		Social Reactivity		Social Support		Error As	Error k
			As	k	As	k	As	k		
England	Males	308	−0.040	−0.985	0.169	−0.776	−0.479	−0.639	0.139	0.277
	Females	301	0.491	−0.656	−0.542	−0.277	−0.545	−0.666	0.140	0.280
Spain	Males	815	0.051	−0.798	−0.282	−0.439	−1.014	0.374	0.086	0.171
	Females	862	0.539	−0.460	−0.812	0.497	−0.843	0.043	0.083	0.166
Chile	Males	154	0.452	−0.143	0.005	−0.530	−0.536	−0.287	0.195	0.389
	Females	94	0.714	−0.153	−0.388	−0.878	−0.226	−0.870	0.249	0.493

**Table 3:** Descriptive statistics.

Country	Gender	N	Aggressive Tendency	Social Reactivity	Social Support
			M ± SD	M ± SD	M ± SD
England	Males	308	5.88 ± 3.06	6.68 ± 3.23	5.97 ± 2.28
	Females	301	4.45 ± 2.88	10.06 ± 2.98	5.67 ± 2.49
	TOTAL England	609	5.17 ± 3.06	8.35 ± 3.54	5.82 ± 2.39
Spain	Males	815	5.37 ± 2.92	8.00 ± 3.08	6.98 ± 2.01
	Females	862	4.20 ± 2.89	11.22 ± 2.56	6.67 ± 2.10
	TOTAL Spain	1677	4.77 ± 2.96	9.66 ± 3.25	6.82 ± 2.06
Chile	Males	154	4.08 ± 2.29	6.99 ± 3.41	5.67 ± 1.99
	Females	94	3.89 ± 2.57	9.19 ± 3.43	5.20 ± 2.05
	TOTAL Chile	248	4.01 ± 2.40	7.82 ± 3.57	5.50 ± 2.02
	TOTAL Males	1277	5.34 ± 2.93	7.56 ± 3.21	6.58 ± 2.14
	TOTAL Females	1257	4.24 ± 2.87	10.79 ± 2.82	6.32 ± 2.26

**Table 4:** Differences in emotional behaviour by country, country × gender, and gender.

VI	VD	Welch	Sig.	ɳ_p_^2^
Country	Aggressive tendency	17.756	0.000	0.011
	Social reactivity	52.365	0.000	0.043
	Social support	75.443	0.000	0.059
Country × gender	Aggressive tendency	27.049	0.000	0.003
	Social reactivity	180.612	0.000	0.003
	Social support	33.223	0.000	0.000
		t	Sig.	ɳ_p_^2^
Gender	Aggressive tendency	9.505	0.000	0.014
	Social reactivity	26.888	0.000	0.118
	Social support	2.967	0.003	0.004

**Table 5:** Post hoc test of differences according to the variable country.

Country (I)	VD	Country (J)	
		Spain (J) Dif I-J (*p*.)	Chile(J) Dif I-J (*p*.)
England (I)	Aggressive tendency	0.404 (0.015)	1.166 (0.000)
	Social reactivity	−1.305 (0.000)	0.529 (0.112)
	Social support	−1.001 (0.000)	0.325 (0.131)
Spain (I)	Aggressive tendency		0.762 (0.001)
	Social reactivity		1.833 (0.000)
	Social support		1.326 (0.000)

**Table 6:** Post hoc test of differences according to the variable country × gender.

VD	(I) Country × Gender	(J) Country × Gender				
		English Females Dif I-J (*p*.)	Spanish Males Dif I-J (*p*.)	Spanish Females Dif I-J (*p*.)	Chilean Males Dif I-J (*p*.)	Chilean Females Dif I-J (*p*.)
Aggressive tendency	English males	1.421 (0.000)	0.507 (0.123)	1.674 (0.000)	1.799 (0.000)	1.983 (0.000)
	English females		−0.914 (0.000)	0.252 (.782)	0.377 (0.652)	0.561 (0.472)
	Spanish males			1.166 (0.000)	1.291 (0.000)	1.476 (0.000)
	Spanish females				0.125 (0.991)	0.309 (0.883)
	Chilean males					0.184 (0.993)
Social reactivity	English males	−3.371 (0.000)	−1.317 (.000)	−4.533 (0.000)	−0.302 (0.943)	−2.506 (0.000)
	English females		2.054 (0.000)	−1.162 (0.000)	3.069 (0.000)	0.865 (0.244)
	Spanish males			−3.216 (0.000)	1.015 (*p* = 0.009)	−1.189 (*p* = 0.021)
	Spanish females				4.231 (0.000)	2.027 (0.000)
	Chilean males					−2.204 (0.000)
Social support	English males	0.296 (0.645)	−1.015 (0.000)	−0.702 (0.000)	0.292 (0.718)	0.765 (0.028)
	English females		−1.312 (0.000)	−0.998 (0.000)	−0.004 (1.00)	0.469 (0.445)
	Spanish males			0.313 (0.022)	1.307 (0.000)	1.781 (0.000)
	Spanish females				0.994 (0.000)	1.467 (0.000)
	Chilean males					0.473 (0.478)

**Table 7:** Correlation between emotional behaviour and effective personality.

	Aggressive Tendency		Social Reactivity		Social Support	
	Spain (*n* = 151)	Chile (*n* = 248)	Spain (*n* = 151)	Chile (*n* = 248)	Spain (*n* = 151)	Chile (*n* = 248)
Academic self-realisation	−0.28 **	−0.13 *	0.18 *	0.19 **	0.18 *	−0.04
Socio-affective self-realisation	−0.04	−0.09	0.23 **	0.16 **	0.41 **	0.05
Resolute efficacy	−0.19 *	−0.08	0.04	0.30 **	0.01	−0.01

** Correlation is significant; at the 0.01 level (bilateral); * Correlation is significant at the 0.05 level (bilateral).

## Data Availability

The datasets generated for this study are available by request from the corresponding author.
